# Impact of COVID-19 on patient and healthcare professional attitudes, beliefs, and behaviors toward the healthcare system and on the dynamics of the healthcare pathway

**DOI:** 10.1186/s12913-021-07237-y

**Published:** 2021-12-06

**Authors:** Katarzyna Bernacki, Angie Keister, Nadia Sapiro, Jin Su Joo, Lisa Mattle

**Affiliations:** 1 Astellas Pharma Inc. Patient Centricity, 1 Astellas Way, Northbrook, IL 60062 USA; 2 Throughline Strategy, Toronto, ON Canada

**Keywords:** Attitudes, Behaviors, Beliefs, COVID-19, Healthcare pathway, Patient feedback, Telehealth, Treatment experience

## Abstract

**Background:**

COVID-19 has dramatically changed how healthcare is delivered and experienced.

**Methods:**

One-on-one interviews and a virtual ethnographic roundtable were conducted among 45 patients, caregivers, and healthcare professionals (HCPs) in 4 therapeutic areas from the United States and Japan: overactive bladder, vasomotor symptoms, prostate cancer, and metastatic urothelial carcinoma. The goal was to identify the impact of COVID-19 on patient/caregiver and HCP attitudes, interactions, beliefs, and behaviors toward the healthcare system and care pathway.

**Results:**

Four foundational themes were identified: 1) COVID-19 risk is relative; 2) isolation is collateral damage; 3) telehealth is a parallel universe; and 4) COVID-19 is destabilizing the foundations of healthcare. Numerous insights, influenced by diverse cultural, social, and psychological factors, were identified within each theme.

**Conclusions:**

The impacts of COVID-19 were noticeable at multiple points of care during the “universal” care pathway, including at initial screening, referral to specialists, diagnosis, treatment initiation/surgery, and during ongoing care. Greater appreciation of the short- and long-term impacts of COVID-19 and resulting gaps in care may act as a catalyst for positive change in future patient care.

**Supplementary Information:**

The online version contains supplementary material available at 10.1186/s12913-021-07237-y.

## Background

COVID-19 has produced an unprecedented disruption in how healthcare is delivered and received. Disruptions include decreased in-person healthcare visits, delays in diagnosis and treatment initiation, increased telehealth [1–7], and changes in treatments and monitoring [6], but they extend beyond these process-related effects. Research suggests that certain social determinants of health (eg, poverty, race, ethnicity, smoking status) may cause disproportionate impacts of COVID-19 on particular groups [8]. The psychological and behavioral impacts of the COVID-19 pandemic on the healthcare environment are only just coming to light. There is a need to understand how patients, providers, and systems are handling the psychological burden of the pandemic and the behavioral responses that impact the way healthcare is experienced. The COVID-19 pandemic has induced changes within the healthcare system, and it is only with better appreciation for the impacts of these changes that further modifications can be integrated into patient care to create a suitable healthcare environment for a postpandemic world.

This study was designed to provide insight and foresight on the impact of COVID-19 on patient/caregiver and healthcare professional (HCP) attitudes, interactions, beliefs, and behaviors toward the healthcare system over both the short- and long-term and to explore the impact of COVID-19 on the broader dynamics of the healthcare pathways, including impacts on decision making, access to care, and mental/holistic health needs.

## Methods

### Aim

The goal was to identify the impact of COVID-19 on patient/caregiver and HCP attitudes, interactions, beliefs, and behaviors toward the healthcare system and care pathway. The research was developed with a comparative design across two geographical regions, the United States (US) and Japan, chosen based on their similarities (eg, COVID-19 impacts of approximately similar magnitudes and timing during the research window) and differences (eg, type of healthcare systems and COVID-19-related public discourse) to enable identification of the role national and local factors played in how the pandemic impacted the care pathway and emotional experience of patients/caregivers.

### Participants

Participants for interviews were recruited by a third-party contractor with access to a large database of patients and HCPs within the US and Japan. Eligible participants were diagnosed with or providing treatment for conditions from 4 therapeutic areas, including 3 chronic conditions (overactive bladder [OAB; Japan only], vasomotor symptoms [VMS; US only], prostate cancer) and 1 acute condition (metastatic urothelial carcinoma [mUC]). Additional screening criteria are provided in Table 1. Caregivers of eligible patients (eg, family member, volunteer, paid helper) could also be included. This research study was performed in accordance with the Declaration of Helsinki and was reviewed and approved by the Astellas Ethics and Compliance committee. All participants provided written informed consent to participate.

**Table 1:** Screening Criteria for Patients and HCPs

Patients	HCPs^**a**^
**VMS** • Age 50–59 years • Experience ≥5 hot flashes/day lasting 10 min • ≥12 months since menstrual period • Willing to see a doctor and take medical treatment	**Oncologists**^**b**^ • Minimum of 15 prostate cancer and mUC patients • Must prescribe infusion therapy
**Overactive bladder** • Willing to see a doctor and try drug treatment • Mix of early-, mid-, and late-stage	**Gynecologists** • Minimum 15 VMS patients
**Prostate cancer** • Age 61–68 years • Must be receiving treatment	**Urologists**^**b,c**^ • Minimum 10 prostate cancer and 5 mUC patients • Must prescribe infusion therapy
**mUC** • Age 51–65 years • Must be receiving treatment • Metastatic (US) and nonmetastatic (Japan) cancer	**Primary care physicians** • Minimum 15 OAB patients

*Abbreviations*: *HCP* Healthcare professional, *mUC* Metastatic urothelial carcinoma, *OAB* Overactive bladder, *US* United States, *VMS* Vasomotor symptoms

^a^HCPs were required to have been in medical practice for at least 2 but no more than 35 years; US HCPs were recruited from states where there were sufficient cases of COVID-19 to impact their practice

^b^Urologists and oncologists were required to treat both prostate cancer and mUC

^c^Urologists were required to have prescribed infusion therapy; US urologists must not work at a government hospital and Japanese urologists must work at an academic hospital or private practice

### Data collection

The primary research was conducted from May 12–June 1, 2020, in the US and May 16–June 15, 2020, in Japan, during which impacts of the COVID-19 pandemic on healthcare were first emerging. Data were collected from double-blinded 60-min, semi-structured, one-on-one phone interviews with patients, caregivers, and HCPs in the US and Japan (*n* = 35), and from a double-blinded virtual ethnographic roundtable with HCPs in the US (*n* = 10). Interviews were performed in the English language in the US and performed in the Japanese language (using the same discussion guide used for US participants) in Japan. Additional methodological details are provided in Supplemental Table 1.

### Data analysis

A grounded theory approach was used (Supplemental Table 1) to review English-language and Japanese-translated transcripts from the interviews and ethnographic roundtable to identify consistent themes regarding the impact of COVID-19 on: 1) patient/caregiver and HCP attitudes, interactions, beliefs, and behaviors toward the healthcare system; and 2) the effect on the “universal” care pathway across the 4 disease states studied.

## Results

### Participants

Twenty-five patients/caregivers from the US (*n* = 13) and Japan (*n* = 12) with the 4 target conditions and 20 HCPs from the US (*n* = 10) and Japan (*n* = 10) who treat these conditions were included (Table 2**)**. US and Japanese HCPs had 8–28 years and 6–31 years in practice, respectively, and spent 80–100% and 80–95%, respectively, of their time in direct patient care.

**Table 2:** Patients, Caregivers, and HCPs

	VMS (US only)	OAB (Japan only)	Prostate Cancer	mUC
**United States**				
Patients (*n* = 10)	*n* = 3		*n* = 3	*n* = 4
Caregivers (*n* = 3)			*n* = 1	*n* = 2
HCPs (*n* = 10)	Gynecologists (*n* = 3)		Urologists^a^ (*n* = 2) Oncologists^a^ (*n* = 5)	
**Japan**				
Patients (*n* = 12)		*n* = 3	*n* = 4	*n* = 5
HCPs (*n* = 10)		PCPs (*n* = 3) Urologists^b^ (*n* = 2)	Urologists^a,b^ (*n* = 6)	

*Abbreviations*: *HCP* Healthcare professional, *mUC* Metastatic urothelial carcinoma, *OAB* Overactive bladder, *PCP* Primary care physician, *US* United States, *VMS* Vasomotor symptoms

^a^Must treat both prostate cancer and mUC. ^b^Could treat both OAB and prostate cancer/mUC

### Findings

Analysis of responses in the interviews and roundtable revealed 2 critical areas that are influenced by COVID-19: 1) foundational insights that shape the human experience of the pandemic within the context of healthcare and 2) the impact of COVID-19 on the healthcare pathway. Four thematic areas of foundational insights that apply globally across therapeutic areas were identified: 1) COVID-19 risk is relative; 2) isolation is collateral damage; 3) telehealth is a parallel universe; and 4) COVID-19 is destabilizing the foundations of healthcare. The impact of COVID-19 was also evidenced at 5 points of care during the “universal” care pathway: 1) initial screening; 2) referral to specialists; 3) diagnosis; 4) treatment initiation/surgery; and 5) ongoing care.

#### Thematic areas of foundational insights

**Fig. 1:**
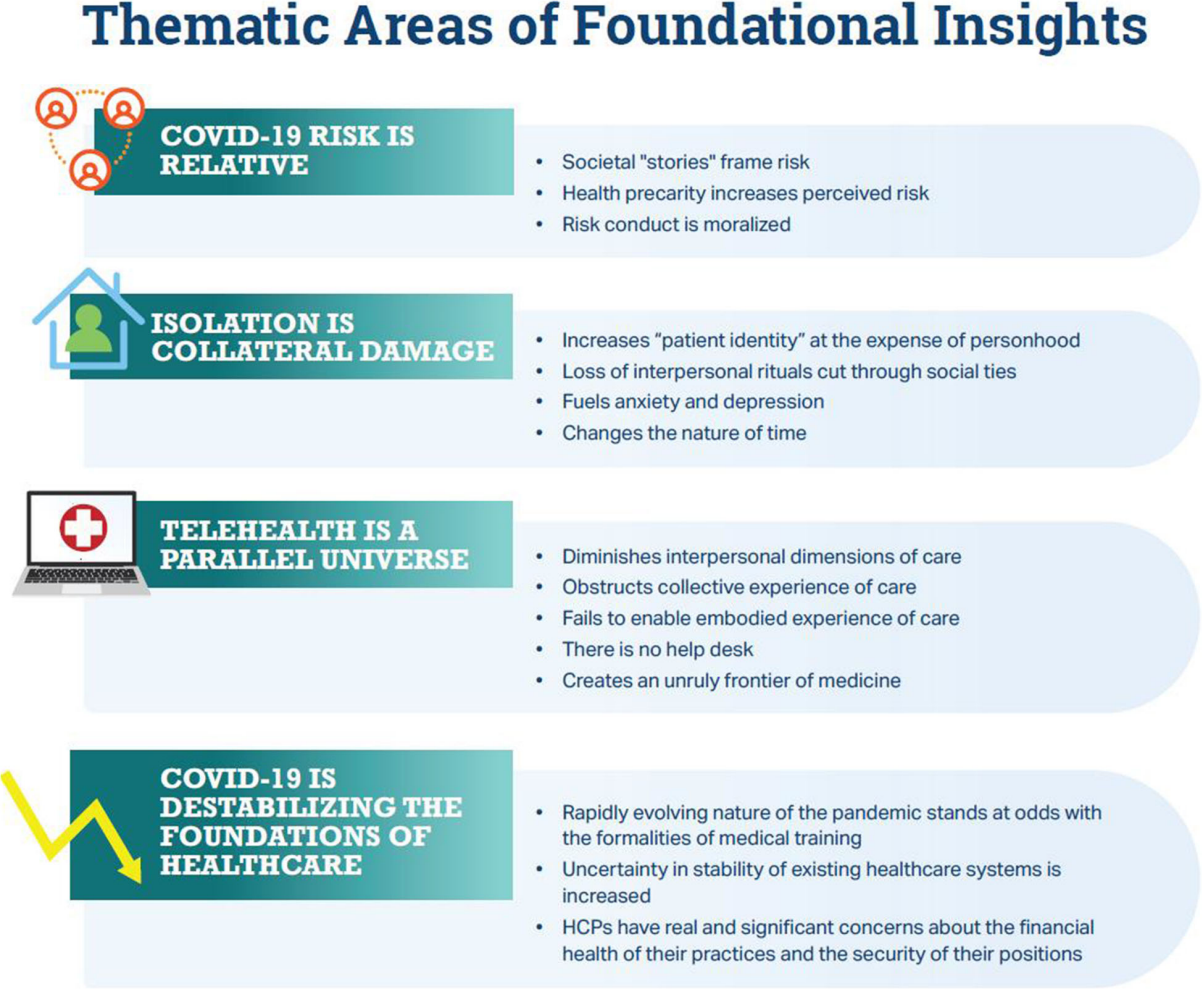
Thematic Areas of Foundational Insights. Abbreviation: HCP, healthcare professional

Within each of the 4 foundational themes identified there are several insights (Fig. 1), which are supported by direct quotes from patients and HCPs (see quotes within text and Supplemental Table 2). These are discussed in depth in the following sections.

#### COVID-19 risk is relative

The perceptions of the risk that the virus carries varied dramatically between individuals and geographic regions due to complex and divergent cultural, social, and psychological factors. Each society forms its own “cultural imaginaries” or societal “stories” that are shaped by the existing value system, media, political policies, religion, etc. [9]. These collective and social ideas, beliefs, and values within a society are a key driver that shapes the perception of risk of COVID-19. The influence of cultural and political contexts on risk perception was highlighted by an mUC patient from the US who attributed political uncertainties of the time to their perception of COVID-19 risk:

*“Every day I turn on the news, I’m hearing different things. The virus is very unpredictable. You don’t know what you’re facing. You don’t know anything. And honestly, I think the higher authority, the governments, even the medical industry are new to this. They don’t know it either.”*

Perceptions of COVID-19 risk were also influenced by how secure or vulnerable people feel about their lives and health. Feelings of vulnerability about health increase the perceived risk and hinge on precarity (ie, feelings of helplessness, displacement, and insecurity) and health locus of control (ie, ability to control/manage health). The impact of precarity was noted by an mUC patient from the US:

*“The virus is affecting people with a compromised immune system, people that are undergoing cancer treatment, people that are older, all the dynamics affecting me, my ethnicity. It affects males more than females.”*

In general, the risk perception of patients from Japan was less impacted by their health status than for US patients because Japanese patients felt less vulnerable regarding their access to healthcare and, as a result, experienced higher health locus of control. This difference was magnified by greater self-isolation among US patients.

Across geographical areas, COVID-19 was viewed in a moral framework shaped by people’s ideas about right and wrong. In both the US and Japan, the behaviors around reducing the risk of COVID-19 were viewed as a moral responsibility. As expressed by a prostate cancer patient from Japan, behaviors are not just about mitigating contagion, but rather about what is right, good, and responsible:

*“When I go to the supermarket, I keep a distance there. When I come home, I wash my hands and I always gargle, too, so I’m taking those measures … I am doing my part. I’ve been washing my hands properly every day for years.”*

Japanese patients tended to believe that it is people’s moral responsibility to protect themselves (ie, shame of being infected), whereas in the US it was less about individual shame and more about a general responsibility to protect those closest to the self/patient.

#### Isolation is collateral damage

While patients were isolating to protect themselves from viral exposure, the isolation had a significant unintended impact on psychology and behavior. Isolation led patients to increasingly focus on their existing medical conditions in the absence of daily life routines. As a result, they felt more like a patient, taking on the “patient identity” at the expense of whole personhood. In general, patients in the US were isolating to a greater extent than patients in Japan due to differences in risk perception as analyzed above, resulting in a greater impact of isolation on psychology and behavior. As a prostate cancer patient from the US stated:

*“I have had a lot of side effects due to treatment; my life right now is just focused on going to daily treatments and coming home. That’s all I do. In the non-COVID world, I would’ve stopped for coffee or lunch after treatment and not felt as much like a patient.”*

The inability of patients, caregivers, and HCPs to engage in their usual interpersonal rituals and social connections led to a substantial increase in loneliness that cannot be replaced by phone and video calls. According to a prostate cancer caregiver from the US:

*“The physical closeness is not quite there. Even when you’re talking on FaceTime or on WhatsApp or Zoom anything, it’s like watching TV. It’s almost like what is showing on the screen is not real and you can be removed from it. So, I don’t talk to my friends anymore.”*

The social distancing and isolation also impaired mental health, as evidenced by an increase in anxiety and depression. Feelings of anxiety and depression were particularly pronounced in patients experiencing menopause. According to a patient with VMS symptoms from the US:

*“Anything that you add on top of [my condition] is going to generate extreme anxiety and worry. And with what’s going on now and I’m alone and having episodes of crying. I’ve resorted to just accepting it.”*

Isolation has also changed the nature of time. While the pandemic slowed the pace of everyday life, the pace of disease progression was not changed, especially for terminally ill patients who felt that they were losing the already limited time they could spend with loved ones. As an mUC patient from the US put it:

*“What if I die? I feel robbed of the limited time I have to enjoy life because my family can’t spend time with me. It’s depressing. It’s devastating, you know, going through this and having all your family makes life a little bit more enjoyable … This is bringing out extreme stress, depression, and anxiety. I can’t get support from loved ones because of the threat.”*

#### Telehealth is a parallel universe

While patients, caregivers, and HCPs spoke about the technological challenges associated with telehealth, this research found that a core challenge of telehealth is cultural in nature. In other words, telehealth requires developing a new set of norms, beliefs, and behaviors (ie, culture) for a virtual exam room versus those of an in-person environment. However, patients, caregivers, and HCPs had not yet developed or internalized the culture of telehealth due to the sudden implementation of virtual medical care. Significant limitations of telehealth were identified in our interviews: 1) the inability to replicate the benefits of physical co-presence; 2) the obstruction of collective team efforts of both HCPs and patients/families; 3) the lack of formalized technical support teams; 4) unequal access due to socioeconomic and educational barriers to entry; and 5) a lack of standardized practices regarding the best use of telehealth and efforts to replicate human connection. The establishment of effective telehealth is particularly difficult when there is no established patient-HCP relationship or when that relationship is weak or strained. As noted by a prostate cancer patient from the US:

*“Telehealth helps that I can actually reach someone and I know they are there and I can see them. But it’s not good enough. It’s inadequate. I don’t think telehealth is a replacement. It’s almost like if you need a haircut and to just look at the barber through a screen is not going to be helpful.”*

While there were concerns about providing good care through telehealth in both the US and Japan, the importance of physical patient/HCP co-presence was considered more important for providing sound clinical judgement in Japan.

#### COVID-19 is destabilizing the foundations of healthcare

Many of the assumptions and institutions of traditional care are being challenged. HCPs are no longer able to rely on their medical training, established protocols, and skills in the rapidly evolving, chaotic, and demanding situation. Many HCPs and patients in the sample questioned whether the existing healthcare system and associated physical infrastructure are sustainable. Further, many HCPs in both the US and Japan were concerned about the long-term economic implications on their practices (Supplemental Table 2).

#### Impact of COVID-19 on the care pathway

**Fig. 2:**
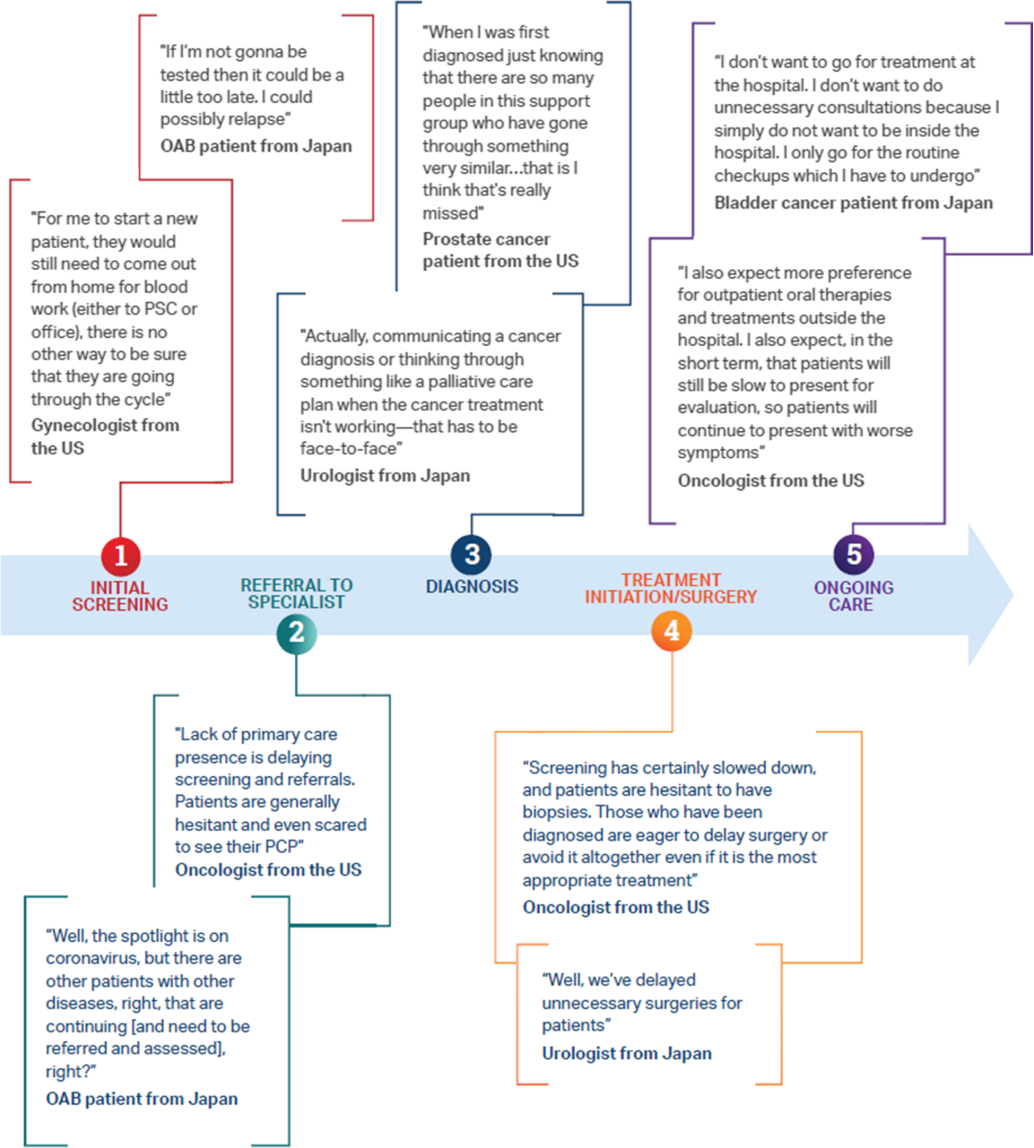
Representative Comments From Participants Regarding Impacts of COVID-19 on Phases of the Healthcare Pathway. Abbreviations: HCP, healthcare professional; OAB, overactive bladder; PCP, primary care physician; PSC, patient service center

In order to understand the impacts of COVID-19 on care pathways across disparate therapeutic areas, 5 phases of care (initial screening, referral to specialists, diagnosis, treatment initiation, and ongoing care) were identified in the interviews as the lowest common denominators universal to most experiences across the 4 therapeutic areas studied. These effects are briefly summarized below with representative patient/HCP comments in Fig. 2**.**

#### Initial screening

The impacts of COVID-19 on screening included deferred primary care visits, avoidance of the emergency department, delays in routine screenings, and limited diagnosis of new conditions. This was particularly pertinent at the time the interviews were conducted (ie, May–June 2020), as participants were still grappling with localized lockdowns and restrictions in access to healthcare facilities. Decreased screening was most prevalent for VMS, OAB, and prostate cancer, with fewer impacts on patients with mUC due to the sense of urgency to initiate treatment.

#### Referrals to specialists

Overall, referrals were disrupted as patients were falling through newly formed cracks between screening and referral. During this early time period, many specialists received fewer referrals from primary care physicians due to lockdowns and reluctance to visit healthcare facilities, and some specialists reported not accepting new patients. The decrease in referrals was most prominent for chronic conditions (ie, menopause/VMS, OAB, prostate cancer) with little change for mUC due to the severity of the condition. Overall, specialists expected to see an increase in referrals once patients resume seeing their primary care physicians.

#### Diagnosis

HCPs considered in-person visits to be critical to proper and timely diagnosis and believed that the benefits of a timely diagnosis outweigh the low risk of COVID-19 infection in healthcare settings. In general, HCPs found it extremely challenging to deliver diagnoses virtually and felt that the roles of caregivers, support groups, and networks (eg, families) were diminished. Some therapeutic areas were more highly affected. For example, gynecologists reported avoiding taking new menopause patients unless patients had access to diagnostic tests for VMS.

#### Treatment initiation/surgery

Without appropriate in-person testing and visits, there was a general reduction in treatment initiation, with the exception for highly progressive and symptomatic conditions (eg, mUC). Treatment decisions were influenced by a desire to minimize preventable infection risk. There were delays in surgery for prostate cancer patients, but less so for mUC patients due to the severity/urgency of the condition. The decline in clinical trial enrollment, both a result of halted trials and patient fear of visiting healthcare facilities, has also influenced treatment decisions. Although new delivery methods have emerged to ensure patients get their medications, barriers still exist for some patients.

#### Ongoing care

The results indicated a dramatic decline in in-person follow-up visits, resulting in greater patient responsibility to self-monitor, identify, and report any issues, which many patients were unprepared to do. Effects on ongoing care included decreased frequency of testing, disruption of infusions/injections, and patient-initiated discontinuations of healthcare-administered treatments. HCPs also expressed reluctance to switch or titrate treatment and difficulty in monitoring toxicity via telehealth. A large discrepancy in telehealth usage, reimbursement, infrastructure, and practices was also noted.

## Discussion

Results of this analysis show that the effects of COVID-19 extend beyond the illness experience into many facets of the human experience. Previous studies have reported that COVID-19 has a significant psychological impact on patients including increased loneliness, fear, and stress and feelings of vulnerability, reduced well-being, and discomfort visiting medical settings [10–15]. Caregivers have been reported to experience fatigue, discomfort, and helplessness, as well as fear, anxiety, and concern for patients and other family members [16]. HCPs have experienced increased vulnerability to infection [17], challenges in providing compassionate care [18, 19], and changes in practice, including navigating the increased use of telehealth and changes in prescribing practices [6, 20–23]. This analysis adds to the existing literature by providing additional insights on how COVID-19 is shaping the illness experience, but also extends these findings to the overall human experience. For patients, caregivers, and HCPs alike, the social, political, and cultural implications of the pandemic have changed the attitudes, beliefs, and behaviors toward the healthcare system and pushed them outside their previously narrow framework.

A key finding of this research was that the perceived risk of COVID-19 is relative, with perceptions of the risk influenced by diverse cultural, social, and psychological factors, often tied to the societal “stories” being told. For example, it was found that Japanese patients have a common social narrative around COVID-19 and feel less vulnerable and more secure about their healthcare access than do US patients. In Japan, there is a culture of positive hygiene practices (eg, mask wearing, hand washing, avoiding crowds and close contact) that likely helps lower risk perception [24]. Japanese patients also feel a moral responsibility to protect themselves and experience greater shame in being infected. For patients in the US, it is less about personal shame and more about a general responsibility to protect each other, especially the elderly.

In support of previous research [14, 15, 25], our findings demonstrate a significant impact of isolation. We found that while patients isolate to protect themselves from the virus, this self-isolation also damages the psyche. There is a loss of personhood and interpersonal rituals, increased anxiety and depression, and a changing nature of time. Since day-to-day routines are disrupted, the notion that someone is a patient rather than an ordinary person is enhanced. In general, Japanese patients felt less fear and isolation than US patients, which is likely related to lower risk perception of COVID-19 stemming from a more cohesive narrative around the virus and a lower sense of vulnerability regarding healthcare access.

As expected, our results from early in the pandemic revealed that the rapidly expanded use of telehealth is changing the nature of how healthcare is delivered. However, since “in-person” norms cannot simply be applied to a digital platform, effective telehealth requires developing a unique culture that is completely different from those established for in-person care. Telehealth cannot replicate the physical co-presence that is believed to be critical in medical care and it obstructs the collective experience of care of both HCP teams and patient support structures. Since transition to telemedicine is more difficult when there is an absence of or a poor patient-HCP relationship, efforts to facilitate stronger relationships can aid in providing effective telehealth services. In addition, telehealth has steep socioeconomic and educational barriers to entry and can exacerbate health disparities that can further increase perceptions of risk and vulnerabilities in socioeconomically disadvantaged groups.

This research further exposed the major impacts of COVID-19 on the delivery of healthcare, including initial screening, specialist referrals, diagnosis, treatment initiation, and ongoing care. Others have reported these trends [1–3, 6, 22, 23], but the current study provides context and key insights from HCPs regarding the impact of these changes on themselves, patients, and the healthcare system as a whole. HCPs were feeling increased insecurity in how they were adapting care for patients. In addition, there have been significant shifts in spaces of care to account for social distancing, including patient screening areas, waiting rooms, examination rooms, infusion clinics, emergency departments, surgical centers, and pharmacies. As local conditions and guidelines shift, care pathways flex and become hyperlocalized. While telehealth has some advantages, there are challenges with providing the cultural and social aspects of care that are more easily achieved with in-person care. As noted by others, substantial effort is required to prevent these negative impacts [20, 21, 26].

During the early stages of the pandemic when this research was conducted, substantial differential effects of COVID-19 on therapeutic area–related diagnosis and treatment were observed. There was little change to the treatment paradigm for an acute and devastating disease such as mUC where the consequences of not treating are dire, whereas chronic therapeutic areas such as VMS and OAB were more significantly impacted because physicians were more focused on managing patients who needed more urgent care and/or because they were not testing/accepting new patients.

While this study provides many new insights into the impact of COVID-19 on the state of healthcare, limitations such as the use of a nonrandom sample and relatively small sample sizes of patients, caregivers, and HCPs should be considered. This small sample may result in feedback that is not necessarily representative of all patients or HCPs within the therapeutic areas evaluated. The qualitative nature of the findings may introduce bias; however, multicoder and multimethod approaches to data collection were implemented to reduce such bias. Importantly, the findings of this study represent a snapshot in time during the early phases of the pandemic and may not be representative of findings obtained at different time points.

## Conclusions

In summary, these results suggest that COVID-19 has had a substantial impact on patient/caregiver and HCP attitudes, interactions, beliefs, and behaviors toward the healthcare system and on the care pathway—both short- and long-term. Future research should focus on identifying methods for healthcare stakeholders to address the pandemic-induced gaps in patient care.

## Supplementary Information

**Additional file 1: Supplemental Table 1.** Methodological Details**. Supplemental Table 2.** Additional Representative Comments Supporting the 4 Foundational Insights.

## Data Availability

The data that support the findings of this research are available on request from the corresponding authors, LM and KB, due to privacy/ethical reasons.

## Abbreviations

HCP: Healthcare professional
mUC: Metastatic urothelial carcinoma
OAB: Overactive bladder
US: United States
VMS: Vasomotor symptoms
