# Supplementary material for: Impact of COVID-19 on patient and healthcare professional attitudes, beliefs, and behaviors toward the healthcare system and on the dynamics of the healthcare pathway
Source: BMC Health Serv Res. 2021 Dec 6;21:1309. doi: 10.1186/s12913-021-07237-y (PMC8646017; doi:10.1186/s12913-021-07237-y)
Supplement: Supplementary file 1 — Additional file 1: Supplemental Table 1. Methodological Details. Supplemental Table 2. Additional Representative Comments Supporting the 4 Foundational Insights. [file 12913_2021_7237_MOESM1_ESM.docx]

Supplemental Table 1. Methodological Details**.**

**Individual interviews**	A discussion guide was developed to ensure consistency in data collection across interviews. The interview flow was the following: respondent introduction, impact of the pandemic on overall life (eg, family, work, etc), impact on condition experience, impact on how they manage and treat the condition, and short-term and long-term outlook. Using open-ended questions, the interviewer allowed the respondents to guide the conversation and probed where necessary. There was 1 interviewer for all interviews in the US and 1 interviewer for all interviews in Japan, with a simultaneous translator.
**Roundtable platform**	Respondents were asked to log in to the roundtable platform every day for 3 days and complete the day's activities (ie, answer questions). Each day's activities were designed such that the respondents were expected to spend about an hour each day, for a total of 3 hours of engagement. Two moderators read their responses each day and posted individual follow-up or clarification questions, which the respondents answered the next day. For shared questions, respondents were able to see other respondents' answers only after they posted their own answers. Respondents were able to interact with others by posting questions or comments to each other's answers. Answers to private questions were not visible to other respondents.
**Data extraction**	A grounded theory approach using both within-case and across-case analysis methods was used. All interviews were transcribed (translated Japanese interviews were transcribed in English) and the VER responses were collected and organized by respondent. A map analytic approach, as described by Carley [1], was used to code the interviews. There were 10 meetings held over a 14-day time period with an average of 5 coders per meeting to review the data collected. Initially, US patients/caregivers, US HCPs, Japan patients, and Japan HCPs were analyzed separately. After initial coding and identification of themes, analyses of the 4 groups were aggregated to identify patterns and points of divergences. Beyond frequent informal discussions, the authors collaborated through 4 formal theming sessions to add, subtract, and refine the findings as part of the iterative analysis process.

Abbreviations: HCP, healthcare professional; US, United States; VER, Virtual Ethnographic Roundtable.

Supplemental Table 2. Additional Representative Comments Supporting the 4 Foundational Insights.

COVID-19 Risk Is Relative	Isolation Is Collateral Damage	Telehealth Is a Parallel Universe	COVID-19 Is Destabilizing the Foundations of Healthcare
"The mass media really started to touch on the fact that famous people are starting to die because of this. Before I didn't think the virus was close to me…I got the sense now that anyone can get this disease" **mUC patient from Japan**	*"I used to wake up to a fairly normal routine. And now it's almost like I have to plan my days or my weeks around radiation treatment. Is there anything else that I'm going to do? All I do and think about is cancer and treatment"* **Prostate cancer patient from the US**	*"When I am with them and I see their facial expression, I feel this sense of trust and I can sense as to whether they are satisfied or not with my treatment. I can tell that by their facial expression, and I think that's really important"* **Generalist from Japan**	*"This is what I imagine war medics feel like because you can only do so much, all the while knowing much of what happens is out of your control"* **Urologist from the US**
"I don't remember worrying so much before this. Now I'm constantly worrying about germs. I'm worrying about germs because I'm worrying about our future financially and about healthcare. Will my husband be able to retire next year? Will I be able to go on trips? If I lose my job, he's obviously going to work a few more years and carry the health insurance for us" **VMS patient from the US**	*"I tend to be a little gabby with people that I like, and so I probably would have stuck around and gotten to know the techs that work on me throughout treatment a little bit more on a personal basis…I think it would have made me feel more like a normal person—it would have made me feel more like myself rather than just patient number 1234. But with COVID-19 the process is to get in and out"* **Prostate cancer patient from the US**	*"One time [Zoom] wasn't working and there wasn't good connection because there were too many people on it at the same time or something. It couldn't handle the volume. My kids couldn't help me, so I had to go to a phone call with my doctor"* **mUC patient from the US**	*"I wonder as to whether we can actually change the healthcare system. I also wonder as to whether we can actually pay for our costs of remote consultation long-term. The system might run out of money"* **Urologist from Japan**

"What's disturbing is there's people that are not wearing masks and they're talking about how they're not bothering anybody. They're increasing the risk for people like me who are at high risk" **mUC patient from the US**	*"Some patients also have more time to be focused or hyperfocused on their bodies. So, they are noticing more symptoms and thinking about their condition"* **Gynecologist from the US**	*"[With remote consultations] there is a high clerical burden. Yet we really don't get a lot out of it, to be honest with you"* **Urologist from Japan**	*"It is very disheartening to work under such stress and put your life and your family's life at risk yet also have to worry about financial survival. We're cutting office hours for some ancillary staff, which will make them quit their jobs. And yet we are tied financially"* **Gynecologist from the US**

Abbreviations: mUC, metastatic urothelial carcinoma; OAB, overactive bladder; US, United States.

Reference

1. Carley K. Coding choices for textual analysis: a comparison of content analysis and map analysis. Sociol Methodol. 1993;23:75-126
